# Differential photoacoustic spectroscopy for flow gas detection based on single microphone

**DOI:** 10.1016/j.pacs.2024.100624

**Published:** 2024-05-29

**Authors:** Lujun Fu, Jiangshan Zhang, Yufeng Pan, Ping Lu

**Affiliations:** a Wuhan National Laboratory for Optoelectronics (WNLO) and National Engineering Research Center for Next Generation Internet Access System, School of Optical and Electronic Information, Huazhong University of Science and Technology, Wuhan 430074, China; b School of Electronic Information and Communications, Huazhong University of Science and Technology, Wuhan 430074, China; c Wuhan OV Optical Networking Technology Co., Ltd., Wuhan 430074, China

**Keywords:** Flow gas detection, Online detection, Differential spectroscopy, Photoacoustic spectroscopy, Single microphone, C_2_H_2_

## Abstract

Differential photoacoustic spectroscopy (PAS) for flow gas detection based on single microphone is innovatively proposed and experimentally demonstrated. Unlike the traditional systems, only one microphone is used to suppress flowing gas noise. Wavelength modulation spectroscopy and second harmonic detection technique are applied in this PAS system with Q-point demodulation for acetylene (C_2_H_2_) gas detection. The experiment is conducted at 1 atm and 300 K. Different concentrations and flow rates of C_2_H_2_ from 0 sccm to 225 sccm are detected by using nitrogen (N_2_) as the carrier gas, which indicates that the system can respond well to flowing gases while maintaining the noise at the same level. The system response time decreases to 3.58 s while the gas velocity is 225 sccm. The detection limit of 43.97 ppb with 1 s integration time and normalized noise equivalent absorption (NNEA) coefficient of 4.0 × 10^-9^ cm^-1^ W Hz^-1/2^ is achieved at the flow rate of 225 sccm. The firstly proposed differential PAS based on single microphone greatly simplifies the system structure for flow gas detection, which provides a novel route for development of PAS with significant practical implementation prospects.

## Graphical abstract

**Figure:**
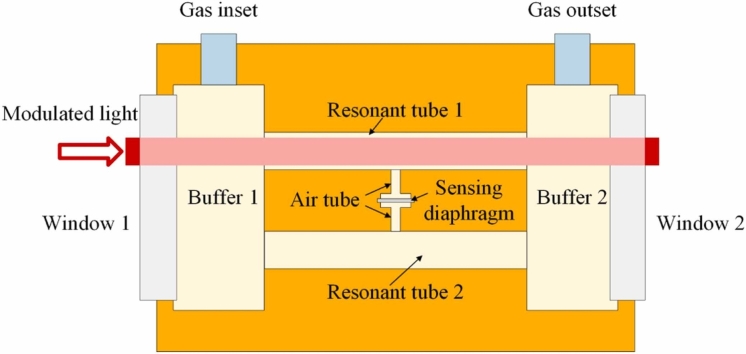

## 1 Introduction

Acetylene (C_2_H_2_), which is a colorless, flammable and explosive gas, is usually dissolved in transformer oil while a specific fault occurs the transformer [1], [2], [3]. And C_2_H_2_ is usually adopted as fault feature for oil-immersed transformer. When discharge fault occurs in oil-immersed electrical equipment, it is usually accompanied by the generation of C_2_H_2_ gas. The concentration of C_2_H_2_ dissolved in the oil can be used as fault diagnosis for the severity of discharge faults occurring in the transformer. Therefore, high-sensitivity detection of C_2_H_2_ is of great significance for early prevention of transformer faults. The traditional detection method for dissolved gas analysis in transformer is gas chromatography (GC) [4], [5]. However, GC has some drawbacks, especially in terms of response time. The analysis results of GC for transformer can’t reflect the real-time situation because of the complicated operation process. Besides, the chromatographic columns need to be replaced periodically, which makes it cumbersome and complex for real time monitoring of transformer. The photoacoustic spectroscopy (PAS) technology is currently being extensively studied due to its advantages such as fast response, high sensitivity and high selectivity [6], [7], [8], [9].

PAS is acknowledged for trace gas detection based on photoacoustic effect [10], [11], [12], [13], [14], [15]. Compared with non-resonant PAS, resonant PAS has the advantage of resonant amplification by the enhancement effect of standing wave [16], [17], [18], [19], [20]. So the resonant PAS usually has higher detection sensitivity. In addition, the working frequency of resonant structures is usually located at the high frequency range, which is less affected by environmental noise. So the impact of external noise can be suppressed. To reduce the impact of external acoustics noise in flow gas detection, differential photoacoustic cell (PAC) structure based on double microphones is applied to detected flow gas [21], [22], [23], [24], [25], [26], [27], [28]. The phases of the photoacoustic signals in the double resonant tubes are opposite and the amplitudes of the signals are the same, while the phases and the amplitudes of external noise are same. Since the microphones have the same performance to acoustic pressure, the noise can be reduced [29], [30], [31], [32]. So the differential PAC can simultaneously test flowing gases and improve the signal-to-noise ratio (SNR) of the PAS system. However, conventional differential PAS requires two matched standard microphones, which increases complexity and equipment costs of the PAS setup. Therefore, a simple PAS system for flow gas detection is urgently worth studying.

In this paper, an all-optical differential PAS for flow gas detection based on single microphone is innovatively proposed and experimentally demonstrated for the first time. Unlike the traditional systems with two matched microphones [21], [28], [29], [31], [32], [33], only one microphone is used to suppress flowing gas noise in the designed system. In the experiment, a distributed feedback laser (DFB) laser with wavelength of 1532.84 nm is used to detect C_2_H_2_. And an erbium-doped fiber amplifier (EDFA) is used to further increase the optical power to 79.8 mW. Wavelength modulation spectroscopy (WMS) and second harmonic (2*f*) detection technique are applied for the system with Q-point demodulation. The experiment is conducted at the environment of 1 atm and 300 K by using N_2_ as the carrier gas. The detection limit of 43.97 ppb with 1 s integration time and normalized noise equivalent absorption (NNEA) coefficient of 4.0 × 10^-9^ cm^-1^ W Hz^-1/2^ is achieved at the flow rate of 225 sccm. Compared with traditional PAS system for flow gas detection, the firstly proposed structure simplifies the system by using only one microphone instead of two matched standard microphones, providing a new route for development of PAS for flow gas detection.

## 2 Theoretical analysis and structure design

**Fig. 1:**
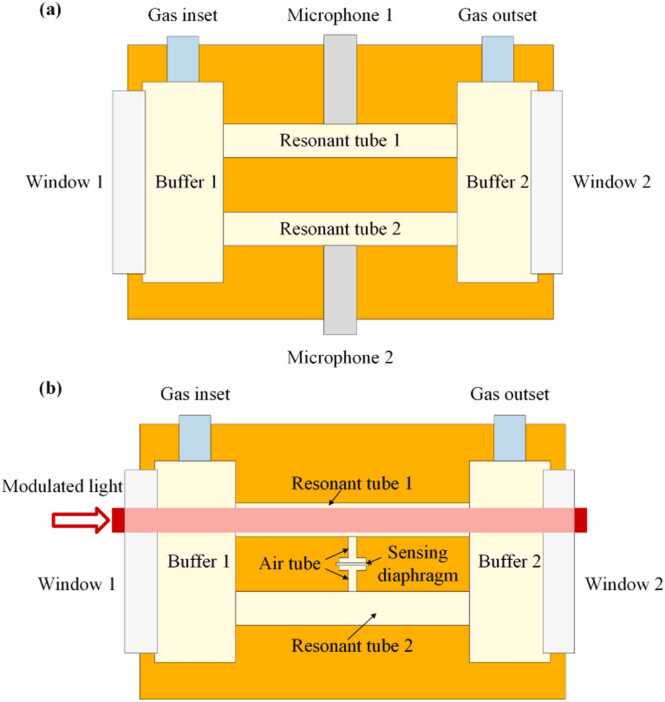
Schematic of **(a)** the conventional differential PAC and **(b)** the designed differential PAC.

Fig. 1(a) shows the conventional differential PAC structure. The PAC consists of two windows, two resonant tubes, two buffers, two microphones, gas inlet and gas outlet. The PAS system works at resonant frequency, so a stable standing wave field is formed in the resonant tubes. Since the PAC structure is symmetrical, the phases of the photoacoustic signals in the two resonant tubes can be opposite while the signal amplitudes are the same. At the same time, the amplitudes and phases of noise are the same in the two resonant tubes because of the symmetrical structure. Therefore, when two standard microphones with the same performance are used to detect the acoustic pressure of the two resonant tubes, the differential signals of the two microphones can increase the signal strength by twice while suppressing noise, which lays the foundation for the detection of flowing gases with PAS technology. The schematic diagram of the firstly designed differential PAC for flow gas detection is shown in Fig. 1(b). Unlike conventional flow gas detection techniques [21], [34], [35], only one microphone is used to detect acoustic pressure. The sensing diagram for acoustic detection is placed in the middle of the two resonant tubes. And two air tubes are used for acoustic conduction. In this firstly designed system, the sensing diagram directly responds to the differential acoustic signal. Only one sensing diagram is applied, which is avoided for the need of two matched standard microphones. This greatly simplifies the complexity of the differential PAS system for flow gas detection.

**Fig. 2:**
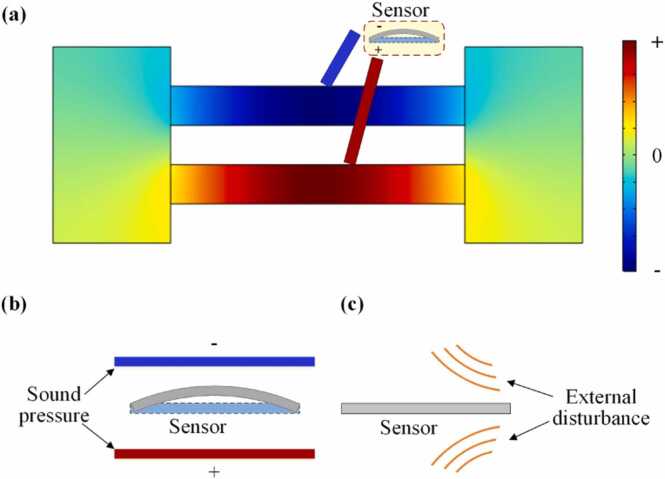
**(a)** Excited mode pressure distribution in the PAC, vibration states of sensor under **(b)** photoacoustic signal and **(c)** external disturbance.

The excited mode pressure distribution in the PAC is shown in Fig. 2(a). The generated photoacoustic signals in the two resonant tubes are out of phase while the amplitudes are the same. As for the external noise in the two resonant tubes transformed from the gas inlet and gas outlet, the noise is in phase and the amplitudes are the same because of the symmetrical structure. In this case, the two photoacoustic signals cause the sensor to move in the same direction, while the two noises cause the sensor to move in opposite direction. Therefore, the sensor only responds to photoacoustic signals while suppressing external noise, as schematically shown in Fig. 2(b) and (c).

**Fig. 3:**
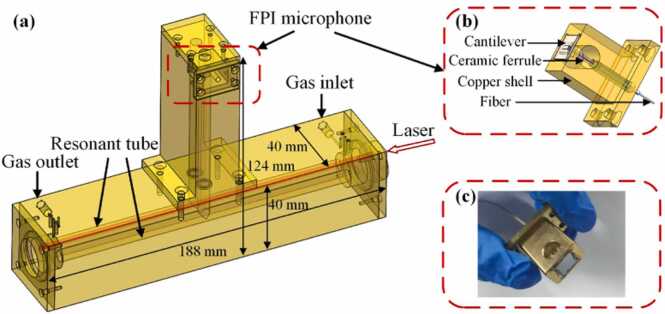
**(a)** The designed differential photoacoustic cell based on single microphone. **(b)** The structural diagram and **(c)** the actual construction of the FPI microphone.

In order to reduce the volume of the PAC and the processing difficulty, the PAC structure is designed, as shown in Fig. 3(a). The length and diameter of the two resonant tubes are optimized to be 6 mm and 160 mm. The spacing between the two resonant tubes is 10 mm. The length and diameter of the two buffers are 18 mm and 10 mm. The length of the two air tubes used for sound conduction is 100 mm. The Fabry-Perot interference (FPI) microphone is placed in the middle of the two air tubes in PAC. The structural diagram and the actual construction of the FPI microphone are shown in Fig. 3(b) and Fig. 3(c), respectively. The FPI microphone consists of a cantilever beam, a ceramic ferrule, a copper shell and a fiber. The performance of the FPI microphone can be referred to the previous work [17]. While PAC operates at the resonant state, two standing waves with opposite phases are generated on the upper and lower sides of the cantilever beam. The noise introduced by the flowing gas at the inlet and outlet can be effectively eliminated after differential treatment with the FPI microphone. Therefore, the detected signal is doubled while suppressing noise. The total chamber volume of the PAC is calculated to be less than 20 mL. The external dimensions are marked in Fig. 3(a). The designed structure can effectively reduce the resonant frequency while increasing the intensity of photoacoustic signal.

## 3 Experimental results and discussions

### 3.1 PAS system design

**Fig. 4:**
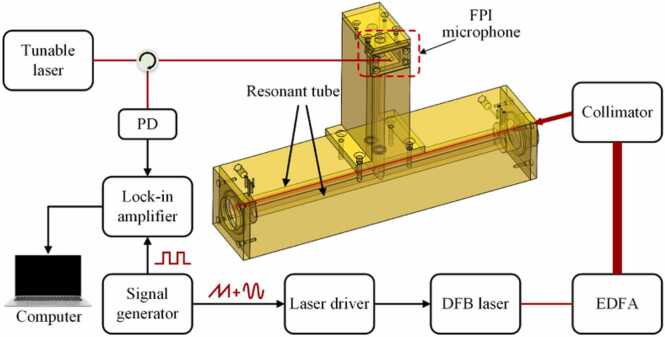
Schematic diagram of the all-optical differential PAS for flow gas detection.

A C_2_H_2_ detection system for flow gas detection is set up based on single microphone. The detailed component of the PAS system is shown in Fig. 4. It includes a DFB laser, an EDFA, a fiber collimator, a laser driver, a signal generator, a tunable laser, a photodetector (PD), an optical fiber circulator, a lock-in amplifier, a computer and a differential PAC. The DFB laser with center wavelength of 1532.84 nm is adopted as excitation light source for C_2_H_2_ gas detection. An EDFA with conventional band (C-band, 1530–1560 nm) is used to increase the optical power of the excitation light source for enhancing the photoacoustic signal. The output light beam is collimated into the PAC module through the fiber-coupled collimator. Wavelength modulation is adopted in the system. A signal generator is used to produce triangular wave superimposed with sine waves, and offer reference frequency for the lock-in amplifier. The triangular wave is used for sweeping the laser wavelength around the gas absorption line, while the sine wave is used for wavelength modulation. The generated photoacoustic signal is received by a FPI microphone. Different harmonic frequency signals are generated while modulating laser wavelength near gas absorption line. The second harmonic signal is selected for demodulation. The Q-point demodulation algorithm is used in the experiment. The photoacoustic signal is collected by the PD and transmitted to the lock-in amplifier for second harmonic demodulation. The experiment is conducted for C_2_H_2_ detection at the pressure of 1 atm and the temperature of 300 K by using N_2_ as the carrier gas.

### 3.2 Experimental results

**Fig. 5:**
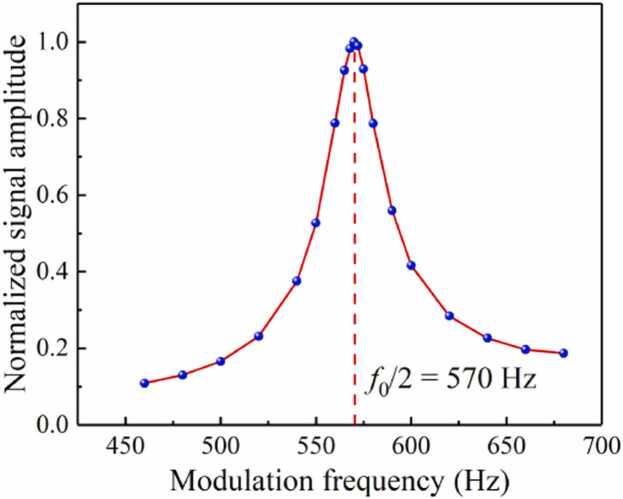
The frequency response of the system.

**Fig. 6:**
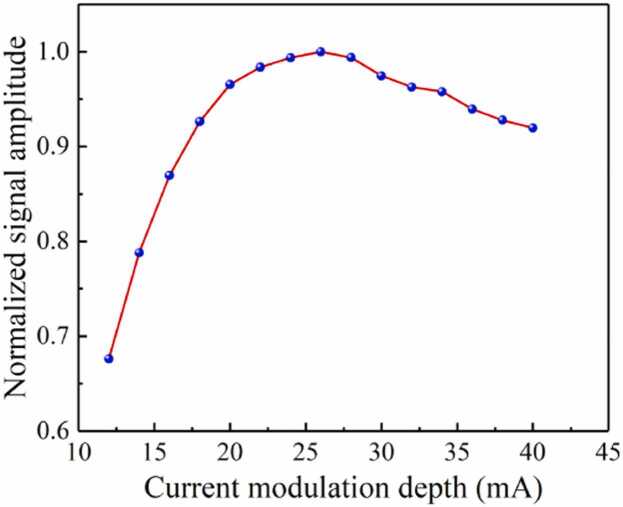
Normalized signal amplitude at different current modulation depths.

To achieve optimal system response, the frequency response curve of the system is obtained by scanning the modulation frequency of the laser. The laser wavelength is fixed at the peak of the C_2_H_2_ absorption line with a constant concentration. The second harmonic signal amplitude is recorded at different modulation frequency. The Fig. 5 shows the normalized signal amplitude as the function of the modulation frequency. The resonant frequency and Q-factor of the system at atmospheric pressure is obtained to be 1140 Hz and 25.4, respectively. The laser modulation depth is another important parameter that must be optimized for achieving optimum photoacoustic signal. The normalized second harmonic signal amplitude at different current modulation depths is obtained at the modulation frequency of 570 Hz, as shown in Fig. 6. The strongest photoacoustic signal is achieved at the modulation depth of 26 mA. Therefore, the wavelength modulation frequency of 570 Hz (*f*_0_/2) and the current modulation depth of 26 mA are selected in the following experiment.

**Fig. 7:**
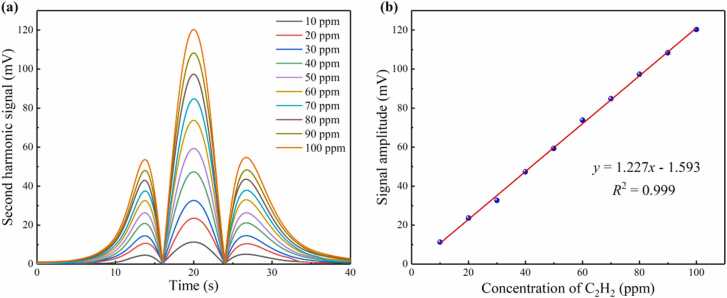
**(a)** The second harmonic signals of C_2_H_2_ with different concentrations and **(b)** linear fitting.

**Fig. 8:**
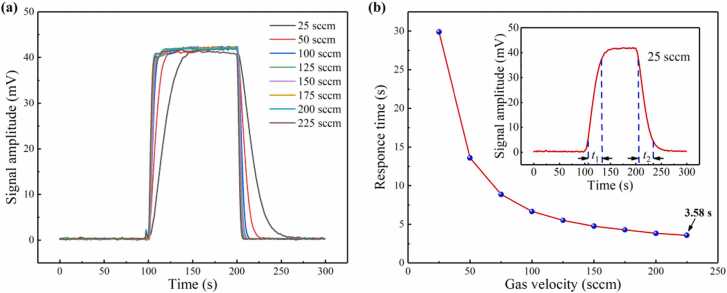
**(a)** System response at the flow rate of 25–225 sccm. **(b)** Relationship between gas velocity and response time.

In order to obtain the system responsiveness and verify the effectiveness of the system on flow gas detection, C_2_H_2_ gases of different flow rates with different concentrations are injected into the PAC. The flow rate and concentration is controlled by the mass flow controller through mixing the standard C_2_H_2_ gas with pure N_2_. Different concentrations of C_2_H_2_ gas from 10 ppm to 100 ppm are measured in turn. The second harmonic photoacoustic signals with different concentration of 10–100 ppm is shown in Fig. 7(a). The relationship between different concentrations of C_2_H_2_ and the second harmonic signals is shown in Fig. 7(b). The linear fitting shows that a slope of 1.227 mV/ppm and an R^2^ value of 0.999 are achieved for the PAS system. The response time is defined as the time required for a signal to rise from 10 % to 90 % or decrease from 90 % to 10 %. The response time can be divided into rising response time *t*_1_ and falling response time *t*_2_, which are almost equal. In the following experiment, falling response time *t*_2_ is selected as the system response time for research. The time-domain response of the system with the same concentration of C_2_H_2_ but different flow rates from 25 sccm to 225 sccm is shown in Fig. 8(a). At high flow rates, the instantaneous flow of gas can produce an impact, which causes small protrusions at the beginning of the response curves. At the flow rate of 225 sccm, the response amplitude is unstable, which is due to the drift of the working point. Therefore, higher flow rates are not validated in this experiment. In order to achieve higher flow rate detection, other demodulation methods need to be used, such as phase demodulation [36], [37]. The relationship between gas velocity and response time is shown in Fig. 8(b), which indicates that the response time is 3.58 s at the flow rate of 225 sccm. The time-domain response at the flow rate of 25 sccm is obtained as the insert in Fig. 8(b).

**Fig. 9:**
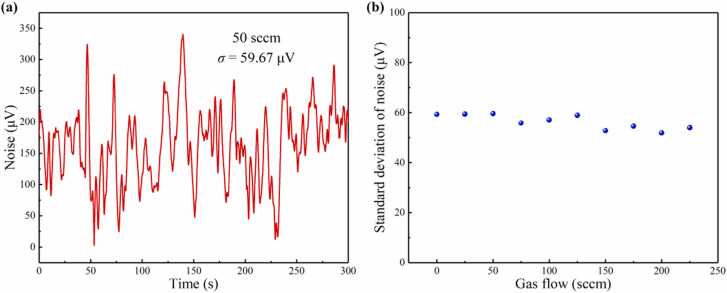
**(a)** The background noise at the flow rate of 50 sccm. **(b)** Relationship between standard deviation of noise and gas velocity.

To obtain noise levels at different flow rates, the noise standard deviation (*σ*) with pure N_2_ filled PAC at different flow rates is recorded, as shown in Fig. 9. Fig. 9(a) shows the background noise at 50 sccm for 300 s, which indicates that the noise standard deviation (*σ*) is 59.67 μV. Fig. 9(b) shows the relationship between standard deviation of noise and gas velocity. The experimental results demonstrate that the noise level remains almost unchanged when the flow rates are between 0 sccm and 225 sccm. Table 1 lists the noise standard deviation (*σ*) and response time (*t*_2_) at different flow rates. According to the responsivity of 1.227 mV/ppm and the background noise of 53.95 μV with the integration time of 1 s, the detection limit of 43.97 ppb is achieved for C_2_H_2_ gas at the flow rate of 225 sccm. A DFB laser of 5.3 mW is used as excitation light for C_2_H_2_ detection. And an EDFA is used to amplify the laser power. The excitation optical power after EDFA of 79.8 mW is obtained with a power meter. Taking the absorption coefficient of 0.57 cm^-1^ for C_2_H_2_ at the pressure and temperature of 1 atm and 300 K into consideration, the NNEA coefficient is calculated to be 4.0 × 10^-9^ cm^-1^ W Hz^-1/2^ at the flow rate of 225 sccm.

**Table 1:** The noise and response time at different flow rates.

Gas flow (sccm)	Noise (*σ*) (μV)	Response time (*t*_2_) (s)
0	59.33	—
25	59.44	29.88
50	59.67	13.60
75	55.85	8.87
100	57.05	6.66
125	58.96	5.51
150	52.76	4.76
175	54.57	4.29
200	51.87	3.84
225	53.95	3.58

## 4 Conclusions

In summary, a novel differential PAS system based on single microphone is firstly designed for flow gas detection. Unlike traditional differential PAS systems with two matched microphones [21], [28], [29], [31], [32], [33], only one microphone is required in this system. WMS and 2*f* detection technique are applied in this PAS system with Q-point demodulation for C_2_H_2_ detection. The experiment is conducted at 1 atm and 300 K by using N_2_ as the carrier gas. Experimental verification with flow rates from 0 to 225 sccm is conducted, which indicates that the system can respond well to flowing gases while maintaining noise at the same level. The system response time decreases to 3.58 s while the gas velocity is 225 sccm. The detection limit of 43.97 ppb with 1 s integration time and NNEA of 4.0 × 10^-9^ cm^-1^ W Hz^-1/2^ is achieved at the flow rate of 225 sccm. However, higher flow rates are not tested in the experiment owing to the drift of working point with Q-point demodulation. This all-optical differential photoacoustic spectroscopy for flow gas detection based on single microphone provides a novel route for flowing gases detection with significant practical implementation prospects. In the following work, the demodulation algorithm or sensor structure can be further optimized and improved for higher flow rates, such as using phase demodulation or using other non-optical microphones.

## CRediT authorship contribution statement

**Ping Lu:** Writing – review & editing, Supervision, Resources, Project administration, Funding acquisition. **Jiangshan Zhang:** Resources, Project administration, Funding acquisition. **Yufeng Pan:** Validation, Supervision. **Lujun Fu:** Writing – review & editing, Writing – original draft, Investigation.

## Declaration of Competing Interest

The authors declare that they have no known competing financial interests or personal relationships that could have appeared to influence the work reported in this paper.

## Data Availability

No data was used for the research described in the article.
